# Anxiety and Depression and their Association with Low Quality of Life in Patients with Metabolic Syndrome

**DOI:** 10.5935/abc.20180221

**Published:** 2018-12

**Authors:** Mariane Lopes da Silva, Mariana Alievi Mari

**Affiliations:** Instituto de Cardiologia/Fundação Universitária de Cardiologia (ICFUC), Porto Alegre, RS - Brazil

**Keywords:** Depressive Disorder, Stress, Physiological, Depression, Metabolic Syndrome, Quality of Life

**Dear Editor,**

We read the article entitled "Lifestyle Intervention on Metabolic Syndrome and its Impact on Quality of Life: A Randomized Controlled Trial", by Saboya et al.^1^ with great interest and would like to contribute with some suggestions.

Firstly, regarding the method. There are no reports about the blinding of the evaluators, both for the interviews and for body mass index and waist circumference measurements. This is a factor considered a high risk of bias by the "Cochrane Collaboration's tool for assessing risk of bias"^2^ since the interviewers, even unconsciously, may influence the responses and their view of the participants.

Secondly, regarding the results. The researchers reported that the quality of life of patients with metabolic syndrome is affected not only by the clinical picture but is also significantly affected by the presence of depression and anxiety.^1^ The prevalence of depression and anxiety was 41.7% and 22.2%, respectively, and these data are not associated with the metabolic syndrome components.

Anxiety and depression have often been associated with metabolic syndrome, as well as other non-transmissible chronic diseases, due to the limiting characteristic the disease has on the individuals' lives. The present study did not demonstrate this association, perhaps due to the sample size, which was too small for a study with so many stages and variables for assessment.

The substantial loss of subjects may have compromised the results. Most studies^3,4^ that associate metabolic syndrome with depression and anxiety, and also evaluate these patients' quality of life, have a larger sample size.
